# ‘PartBreCon’ study. A UK multicentre retrospective cohort study to assess outcomes following PARTial BREast reCONstruction with chest wall perforator flaps

**DOI:** 10.1016/j.breast.2023.07.007

**Published:** 2023-07-17

**Authors:** A. Agrawal, L. Romics, D. Thekkinkattil, M. Soliman, M. Kaushik, P. Barmpounakis, C. Mortimer, C.A. Courtney, A. Goyal, E. Garreffa, A. Carmichael, R.A. Lane, C. Rutherford, B. Kim, R. Achuthan, V. Pitsinis, S. Goh, B. Ray, K. Grover, R. Vidya, J. Murphy, PartBreCon Collaborators, Dorin Dumitru, Raouef Bichoo, Nirbhaibir Singh, Hussein Tuffaha, Evangelos Mallidis, Kalliope Valassiadou, Venla Kantola, Lydia Prusty, Anzors Gvaramadze, Vivienne Blackhall, James Mansell, Ahmed Hamad

**Affiliations:** s Dorin Dumitru, Hull Royal Infirmary, Hull; t Raouef Bichoo, Hull Royal Infirmary, Hull; u Nirbhaibir Singh, Royal Wolverhampton NHS Trust, Wolverhampton; v Hussein Tuffaha, Ipswich Hospital, Ipswich; w Evangelos Mallidis, Ipswich Hospital, Ipswich; x Kalliope Valassiadou, University Hospitals of Leicester NHS Trust, Leicester; y Venla Kantola, St. James’s University Hospital, Leeds; z Lydia Prusty, Lincoln County Hospital, Lincoln; aa Anzors Gvaramadze, Lincoln County Hospital, Lincoln; ab Vivienne Blackhall, Gartnavel General Hospital, Glasgow; ac James Mansell, University Hospital Wishaw, Wishaw; ad Ahmed Hamad, Royal Derby Hospital, Derby; a Cambridge University Hospitals, Cambridge, UK; b New Victoria Hospital, Glasgow, UK; c Lincoln County Hospital, Lincoln, UK; d Mansoura University, Egypt; e University Hospitals of Leicester NHS Trust, Leicester, UK; f Department of Statistics, Athens University of Economics and Business, Athens, Greece; g Ipswich Hospital, Ipswich, UK; h Royal Derby Hospital, Derby, UK; i University Hospital of Derby and Burton, Belvedere Road, Burton on Trent, UK; j Gartnavel General Hospital, Glasgow, UK; k St. James's University Hospital, Leeds, UK; l Ninewells Hospital, Dundee, UK; m Peterborough Hospital, Peterborough, UK; n Harrogate NHS Trust, Harrogate, UK; o Hull Royal Infirmary, Hull, UK; p Royal Wolverhampton NHS Trust, Wolverhampton, UK; q Manchester University Hospital, Manchester, UK; r PartBreCon Collaborators, UK

**Keywords:** Oncoplastic breast surgery, Partial breast reconstruction, Chest wall perforator flap, LICAP, LTAP, TDAP

## Abstract

**Background:**

Partial breast reconstruction with a pedicled chest wall perforator flap (CWPF) enables breast conservation in a higher tumour: breast volume ratio scenario. Since there is limited evidence, this retrospective cohort study aimed to ascertain immediate (30-days) and medium-term (follow-up duration) surgical outcomes.

**Methods:**

STROBE-compliant protocol ascertained CWPF outcomes between March 2011–March 2021. UK centres known to perform CWPF were invited to participate if they performed at least 10 cases. Data were retrospectively collected, including patient demographics, tumour and treatment characteristics, and surgical and oncological outcomes. Statistical analysis (R™) included multivariable logistic regression and sensitivity analysis.

**Results:**

Across 15 centres, 507 patients with median age (54 years, IQR; 48–62), body mass index (25.4 kg/m^2^, IQR; 22.5–29), tumour size (26 mm, IQR; 18–35), and specimen weight (62 g, IQR; 40–92) had following flap types: LiCAP (54.1%, n = 273), MiCAP/AiCAP (19.6%, n = 99), LiCAP + LTAP (19.8%, n = 100) and TDAP (2.2%, n = 11). 30-days complication rates were in 12%: haematoma (4.3%, n = 22), wound infection (4.3%, n = 22), delayed wound healing (2.8%, n = 14) and flap loss (0.6%, n = 3; 1 full) leading to readmissions (2.6%, n = 13) and re-operations (2.6%, n = 13). Positive margins (n = 88, 17.7%) led to 15.9% (n = 79) re-excisions, including 7.5% (n = 37) at the planned 2nd of 2-stage surgery and 1.8% (n = 9) mastectomy. At median 23 months (IQR; 11–39) follow-up, there were 1.2% (n = 6) symmetrisations; recurrences: local (1%), regional/nodal (0.6%) and distant (3.2%).

**Conclusions:**

This large multicentre cohort study demonstrates acceptable complication and margin re-excision rates. CWPF extends the range of breast conservation techniques. Further studies are required for long-term oncological outcomes.

## Highlights

• **PartBreCon study**: A retrospective cohort study of partial breast reconstruction.
• 15 centres, n = 507; T2 (26 mm), Breast conservation in 73.4% A-C cups, BMI (25.4).
• Chest Wall Perforator Flaps - LiCAP (54%), AiCAP (19%), LiCAP-LTAP (20%), TDAP (2%).
• Shows acceptable complications (12%) - re-operations (2.6%), flap loss (0.6%).
• Low Mastectomy (2%) & Low Symmetrisation (1%); FU (23 m), local recurrence (1%).

## Mini-Abstract

This is the analysis of the largest aggregated cohort of patients (n=507, 2011-2021, 15 UK centres) undergoing partial breast reconstruction with a pedicled chest wall perforator flap to avoid deformity following breast conservation. Data demonstrates its applicability in T1-T2 tumours (median size,26 mm, IQR;18-35) with re-excision rates of 15.9 (7.5% during the planned second stage) and 1.8% completion mastectomy. Postoperative complication rates were 4.3% haematoma, 4.3% wound infection, 2.8% delayed wound healing, and 0.2% total flap loss. This study demonstrates that CWPF is a valuable option for patients with a higher tumour-to-breast volume ratio who wish to pursue breast-conserving surgery.

## Graphical abstract

**Figure:**
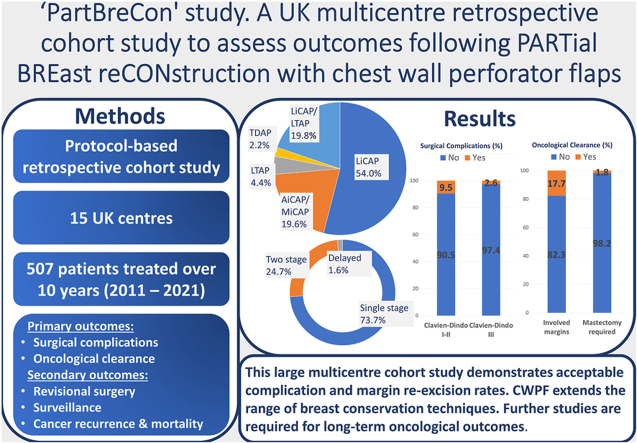

## 1 Background

Oncoplastic breast-conserving surgery (BCS) enables the resection of large tumours, which would otherwise require mastectomy [1] and allows for maintained or improved aesthetics over non-oncoplastic BCS. Volume displacement oncoplastic BCS combines oncological resection with mastopexy and/or reduction mammaplasty. Volume replacement techniques enable partial breast reconstruction following a 20–50% volume loss [2,3]. Partial breast reconstruction inherent to the technique maintains native breast volume and shape without contralateral surgery (in contrast to mammaplasty). The Latissimus Dorsi Mini flap enables partial reconstruction, though with higher post-operative issues, including pain due to muscle cut [4,5]. More recently, chest wall perforator flaps (CWPF) have been used to enable muscle-sparing partial reconstruction avoiding muscle-related issues.

CWPF are pedicled flaps raised on perforating vessels around the breast border. Individual flaps are named after the parent vessel from which each perforator arises according to well-published anatomical landmarks [6,7]. The flaps are MiCAP (Medial intercostal artery perforator), AiCAP (Anterior intercostal artery perforator), LiCAP (Lateral intercostal artery perforator), LTAP (Lateral Thoracic artery perforator) and TDAP (Thoraco-Dorsal artery perforator).

Although CWPF for partial breast reconstruction were described early in the millennium [6,8], their adoption is more recent due to the learning curve and skills needed for the concept, as well as the challenging dissection of small-bore vessels that are smaller than Latissimus Dorsi pedicle. The current CWPF literature is limited to single-centre or small case series with limited clinical outcomes [7,[9], [10], [11], [12], [13]]. A recent systematic review (1990–2020) of 11 studies with 432 cases [14] showed 12.3% overall complication rates.

Therefore, we conducted a retrospective UK multicentre cohort study (PartBreCon study) with the aim to evaluate the surgical outcomes, both immediate (within 30 days of operation) and medium-term (at the median duration of study follow-up) of CWPF partial breast reconstructions following BCS for early breast cancer. This 'PartBreCon' study paper will elaborate on early practice experience on the applicability and probability of having post-operative complications and associate them with patient characteristics and surgical variables.

## 2 Methods

### 2.1 Main outcomes and measures

A) Demographics and Tumour characteristics
  1. Patient demographics: age, body-mass index (BMI), comorbidities
  2. Preoperative tumour characteristics and location influencing surgical planning
B) Treatment characteristics
  1. Surgical: operative data, including flap types and distribution
  2. Oncological: systemic therapies (adjuvant and neoadjuvant), radiotherapy
C) Primary outcome: Surgical
  1. Complications
  2. Oncological clearance: Re-excision rates, conversion to mastectomy
D) Secondary outcomes
  1. Revisional surgery
  2. Surveillance
  3. Oncological: Recurrence and Mortality

### 2.2 Study design, setting, participants and exposure

Since CWPF is a relatively newer application in BCS [6], only a small proportion of UK breast units performed this procedure during the study concept phase (2019–20). Therefore, centres in the UK known to perform CWPF reconstructions were invited. Centres that volunteered were required to have performed a minimum of 10 CWPF to demonstrate experience beyond the early learning phase that could influence surgical outcomes. Patients at each centre were offered all options (simple wide local excision, therapeutic mammaplasty, mastectomy with or without immediate whole breast reconstruction) in keeping with UK oncoplastic guidelines [15]. We collected data on consecutive patients in each centre according to the prospectively maintained local database on CWPF surgery, and this reduces selection bias.

**Inclusion criteria:**

- Patients undergoing partial breast reconstruction using CWPF for primary breast cancer between March 2011–March 2021
- Delayed correction of breast deformity following previous BCS
- Centre to have performed minimum 10 CWPFs

**Exclusion criteria:**

- Patients undergoing volume displacement BCS
- Patients undergoing simple wide local excision (without planned CWPF partial breast reconstruction)
- Patients undergoing mastectomy ± immediate breast reconstruction

### 2.3 Surgical technique

CWPF was performed either by an oncoplastic breast surgeon alone or jointly with a plastic surgeon, according to the published anatomical landmarks and operative steps [3,6,7,16]. In a single-stage procedure, once the cancer resection was completed, the CWPF was raised as a turnover flap (folded 180°), a pendulum type flap based on longer pedicles (TDAP/LTAP) or as a propeller flap (with skin replacement) to reconstruct the tumour excision defect.

A drain was used based on individual intra-operative circumstances (e.g., simultaneous axillary node clearance). This was placed across the donor site and the breast cavity if used. Alternatively, patients underwent a ‘two-stage’ approach if there was a concern regarding achieving clear margin status (e.g., DCIS or invasive lobular cancer). This latter approach involved initial cancer resection filling the resection cavity with water/saline. Patients returned within 4–6 weeks for second-stage partial breast reconstruction [17].

Before the UK Association of Breast Surgery consensus for adopting and accepting 1 mm tumour resection margin in 2015 [18], individual centres' policies varied (mainly between 2 mm and 5 mm); hence, margin distance could not be analysed. Thus, the presented data includes each centre's stated margin status, positive or clear.

### 2.4 Follow-up

The current follow-up policy in the UK National Health Service is annual bilateral mammograms for at least the first five years, followed by a reversal to the 3-yearly National Health Screening programme in women between the age of 50–70. Compared to the previous annual clinical follow-up, the practice has evolved into a patient-led follow-up with an open-door policy to allow women to report directly to the treating unit if there are any symptomatic concerns. The patient would undergo a formal triple assessment if there were a suspicious recurrence, either on mammograms or a symptomatic presentation.

### 2.5 Data management

Each centre lead received local clinical governance authority approval to retrospectively collect anonymised data relevant to the study objectives. Agreed Protocol-based data variables were then entered into Microsoft™ Excel sheet. Participating units securely stored a local spreadsheet linking the study identification number with patient identifiers for cross-checking data, which may be necessary, per Caldicott's principles [19]. No identifiable patient data was centrally submitted or stored.

### 2.6 Statistical methods

Data were analysed using the statistical software R™ (version 4.1.1, www.r-project.org). Descriptive statistics for each variable included counts and percentages of categorical data, whereas median and inter-quartile range (IQR) were calculated for continuous data. Statistical significance was determined using standard Wald tests and the default method in the R™. The statistical significance threshold was considered at 5%. Shapiro-Wilk test was used to test for the normality of the distribution of cases across 15 centres.

Multivariable logistic regression was performed for possible predictors of postoperative events needing intervention (aspirable seroma and complications). A separate sensitivity analysis was performed, including BMI in the best-fit models. The analysis commenced using all variables and continued using backward elimination or forward selection as appropriate, removing or selecting variables aiming for the model with the best Akaike information criterion (AIC). The AIC was chosen as a criterion that deals with the risk of overfitting (by penalising the number of variables selected) and underfitting by performing a trade-off between the model's goodness of fit. Also, the model chosen by leave-one-out cross-validation is asymptotically equivalent to the model selected by AIC. AIC is primarily used in cases where the goal is prediction. We performed a complete case analysis. Patients with any missing data on the possible predictors were assumed missing completely at random and excluded from the analysis to avoid imputation that could possibly introduce bias. The study is reported in line with the Strengthening the Reporting of Observational Studies in Epidemiology (STROBE) guidelines [20].

## 3 Results

507 patients underwent partial reconstruction using CWPFs over ten years (March 2011–March 2021) across 15 centres in the UK (13–107 cases; p = 0.002) with a median follow-up period of 23 (IQR; 11–39) months from the date of surgery. In the first five years (March 2011–March 2016), there were 86 procedures (17%), and in the latter half (April 2016–March 2021), there were 421 procedures (83%). This increase in the use of CWPF was demonstrated across all 15 centres. The spread of cases (107, 47, 47, 45, 39, 34, 34, 32, 20, 19, 19, 18, 17, 16, 13) centre-wise was non-normal distribution.

### 3.1 Demographics and Tumour characteristics

#### 3.1.1 Patient demographics

The median patient age was 54 (IQR; 48–62 years). 39.2% (n = 156/398) were diagnosed with screen-detected breast cancer, whilst 60.8% (n = 242/398) were symptomatic.

The median BMI (kg/m^2^) in 357 available data was 25.4 (IQR; 22.5–29). Breast/bra-cup size data are shown in Table 1a. Other aesthetic data variables usually included during the oncoplastic assessment [21], such as ptosis and skin quality, were not included in our analysis due to insufficient data.

**Table 1a:** Bra-cup size.

Cup	Number	Total (excluding missing values, 251)	Percent
A (including A-B)	33	256	12.8%
B (including B–C)	96	256	37.5%
C (including C-D)	59	256	23%
D	38	256	14.8%
D+	30	256	11.7%

**Table 1b:** Smoking and co-morbidities.

	Yes	No	Missing values	Total (Excluding missing values)	Percent
Smoking	55	425	27	480	11.5%
Comorbidities	127	343	37	470	27%

Comorbidities included diabetes, hypertension, asthma, cardiac conditions, haematological disorders, chronic kidney disease, chronic obstructive pulmonary disease, cerebrovascular accident, connective tissue diseases, deep vein thrombosis/pulmonary embolism, significant autoimmune or neuromuscular disease, or morbid obesity.

Table 1b shows that 11.5% (n = 55) of patients smoked within the previous three months, and 27% (n = 127) had comorbidities, including 4.3% (n = 22) with diabetes.

#### 3.1.2 Preoperative tumour characteristics

The median tumour size in the largest diameter was 26 mm (IQR; 18–35), based on the maximum size of any imaging modality (mammogram, tomogram, ultrasound, or MRI). Sizing can differ between imaging modalities [22]; however, excision planning is around the largest confirmed size. Table 2 shows the preoperative tumour characteristics and location across breast quadrants.

**Table 2:** Preoperative tumour characteristics.

Variable	Number & percentages
**Clinical T staging**	**489**^a^
cTis	54 (11%)
cT1	160 (32.7%)
cT2	254 (51.9%)
cT3	21 (4.3%)
**Tumour type**	**497**^a^
NST	328 (66%)
ILC	63 (12.7%)
Mixed/others^b^	40 (8%)
Benign/borderline^c^	4 (0.8%)
DCIS	62 (12.5%)
**Tumour position**	**458**^a^
UOQ	235 (51.3%)
UIQ	15 (3.3%)
LOQ	107 (23.4%)
LIQ	50 (10.9%)
Central	19 (4.1%)
Others^d^	29 (6.3%)
Multicentric (2 tumours more than 5 cm apart)	3 (0.7%)
**Clinical N staging**	426^a^
cN0	358 (84%)
cN1	68 (16%)

NST, not otherwise specified; ILC, invasive lobular carcinoma; DCIS, ductal carcinoma in situ.

UOQ, upper outer quadrant; UIQ, upper inner quadrant; LOQ, lower outer quadrant; LIQ, lower inner quadrant.

a Total number of cases excluding missing values.

b Mixed/others include 37 mixed pathologies (e.g., NST + ILC), 1 DCIS with microinvasion, 1 giant cell sarcoma and 1 malignant phyllodes.

c Benign/borderline includes 1 benign phyllodes, 1 borderline phyllodes, 1 intraductal papilloma and 1 recurrent papillomatosis.

d Other locations e.g., upper/lower central regions (exactly 6 or 12 o'clock position) and tumours at the inframammary fold.

### 3.2 Treatment characteristics

#### 3.2.1 Surgical: operative data, including flap types and distribution

**Fig. 1:**
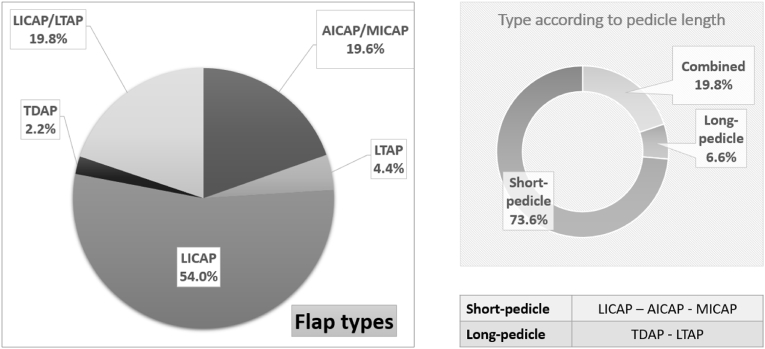
Types of Flap performed.

86% (n = 435) of operations were performed by oncoplastic breast surgeons, and 14% (n = 71) jointly with plastic surgeons. 65.9% (n = 220) were turnover CWPF flaps, 32.6% (n = 109) were propeller flaps, and the remaining 1.5% (n = 5) were croissant flaps (n = 4) or V–Y advancement flaps (n = 1). Table 3 and Fig. 1 show operation and flap types.

**Table 3:** Operation and Flap types.

Variable	Numbers & percentages
**Flap type**	505^a^
LICAP	273 (54%)
LTAP	22 (4.4%)
AICAP/MICAP	99 (19.6%)
TDAP	11 (2.2%)
LICAP + LTAP	100 (19.8%)
**Stages of Surgery**	506^a^
Single	373 (73.7%)
Two	125 (24.7%)
Delayed	8 (1.6%)
**Axillary surgery**	487^a^
None (In-situ disease)	43 (8.8%)
SNB/ANS	363 (74.5%)
ANC	67 (13.8%)
ANC following SNB	14 (2.9%)

LICAP, lateral intercostal artery perforator flap; LTAP, lateral thoracic artery perforator flap.

AICAP/MICAP, anterior/medial intercostal artery perforator flap; TDAP, thoracodorsal artery perforator flap; SNB, sentinel node biopsy; ANC/ANS, axillary node clearance/sampling.

a Total number of cases excluding missing values.

14.6% (n = 74) underwent axillary node clearance, of which 12.3% were performed upfront for positive nodes at diagnosis. Two-stage surgery was performed in 24.7% (n = 125) due to: DCIS 17.6% (n = 22), invasive lobular cancer 10.4% (n = 13), multifocal invasive cancer 16% (n = 20), invasive ductal cancer 56% (n = 70). The proportion of patients undergoing two-stage surgery decreased from 32% to 18% from the first to second half of the study.

1.6% (n = 8) of flaps were utilised in the delayed correction of breast deformity following BCS with defects possibly associated with post-radiotherapy shrinkage.

#### 3.2.2 Oncological: systemic therapies (adjuvant and neoadjuvant), radiotherapy

##### 3.2.2.1 Chemotherapy

44.7% (n = 218) received chemotherapy (neoadjuvant, 13.2%; adjuvant, 31.5%). 12.5% (n = 49) received anti-HER2 treatment. Multigene array analysis supported the decisions regarding chemotherapy use in 71 patients.

##### 3.2.2.2 **Radiotherapy**

96.1% received adjuvant Radiotherapy, and 30.9% received a boost. Radiotherapy was omitted in 3.9% (n = 19) due to patient refusal or participation in Radiotherapy de-escalation trials evaluating the exclusion of Radiotherapy in low-risk diseases.

##### 3.2.2.3 **Endocrine therapy**

Only ten patients received neoadjuvant hormonal therapy, whereas 75.3% of the women received adjuvant endocrine therapy.

### 3.3 Primary outcome: Surgical

40.2% (182 of 453 available) of the study cohort of 507 patients were discharged on the same day (not a 23-hours stay). Drains were used in 36.9% (176/477) with a median duration of 2 days. Seroma without associated complication needing needle aspiration was seen in 3.7% (n = 19), and all were in the flap donor site.

#### 3.3.1 Complications

Overall, 12% (n = 61) patients experienced a complication (excluding seroma); 9.5% (n = 48) were classed as Clavien-Dindo (CD) I-II and 2.6% (n = 13) as CD III. In the first half of the study period (2011–2016), overall complications were 12.8% (11/86) and in the latter half (2016–2021), 11.9% (50/421). There were no surgical-related deaths (within 30 days).

Complications included haematoma (4.3%, n = 22), wound infection (4.3%, n = 22), delayed wound healing (2.8%, n = 14) and 0.6% flap loss (n = 3). Readmissions were in 2.6% (n = 13): infection needing IV antibiotics (n = 7), haematoma (n = 5), and flap loss (n = 2). Unplanned returns to the theatre were in 2.6% (n = 13): for infection (n = 5), haematoma (n = 8), and flap loss (n = 2). Notably, there were only 3 (0.6%) flap losses, one total and two partial. All were before radiotherapy and managed by surgical debridement.

There were no significant associations between postoperative events needing intervention and comorbidities (p = 0.42) or smoking status (p = 0.35). Flap type (propeller vs turnover; p = 0.66), tumour position (inner vs outer quadrants; p = 0.07), and single vs two-stage procedures showed no significant association with complication rates (p = 0.62).

In the multivariable analysis, the largest tumour size (on any imaging modality) was not statistically significant in the full model or the AIC selection method. Neither usual patient risk factors [co-morbidities (RR, 1.06; 95% CI, 0.44–2.47; p = 0.902) and smoking (RR, 1.84; 95% CI, 0.47–6.29; p = 0.359)] nor procedure-specific risk factors [flap type, propeller vs turnover (RR, 1.26; 95% CI, 0.43–3.61; p = 0.666), tumour position (e.g., inner vs outer (RR, 0.283; 95% CI, 0.07–1.13; p = 0.071), single or two stages (RR, 1.502; 95% CI, 0.30–6.66; p = 0.601)] were significantly associated with complications. The only significant factor associated with a lack of complications was the absence of axillary surgery (RR, 52.212; 95% CI, 3.10–1270.02; p = 0.009).

#### 3.3.2 Oncological clearance: Re-excision rates, Conversion to mastectomy

Table 4 shows the postoperative tumour characteristics. In DCIS with available grades (n = 50), the majority were high-grade (84%).

**Table 4:** Postoperative tumour characteristics.

Postoperative tumour characteristics	Numbers & percentages
**Pathological T staging**	492∗
p/ypT0	8 (1.6%)
p/ypTis	65 (13.2%)
p/ypT1	178 (36.2%)
p/ypT2	228 (46.3%)
p/ypT3	13 (2.6%)
**Pathological N staging**	441*
p/ypN0	302 (68.5%)
p/ypN1	103 (23.4%)
p/ypN2	33 (7.5%
p/ypN3	3 (0.7%)
**Grade (invasive tumour)**	430∗
1	52 (12.1%)
2	236 (54.9%)
3	142 (33%)
**Tumour Receptor Status**	
ER positive	371/425 (87.3%)
PR positive	206/289 (71.3%)
HER2 positive	55/350 (15.7%)
**Grade (DCIS)**	50∗
Low	0 (0%)
Intermediate	8 (16%)
High	42 (84%)
**Tumour focality**	494∗
Unifocal	391 (79.1%)
Multifocal/Multicentric	103 (20.9%)
**Margins**	496∗
Clear	408/496 (82.3%)
Involved	88/496 (17.7%)
**Re-excision**	496∗
Yes	79/496 (15.9%)
No	417/496 (84.1%)
**Mastectomy**	499∗
Yes	9/499 (1.8%)
No	490/499 (98.2%)

DCIS, Ductal Carcinoma In Situ; ER, Oestrogen Receptor; PR, Progesterone Receptor; HER2, Human Epidermal Receptor 2.

∗ Total number of cases excluding missing values.

Clear margins were achieved in 82.3% (n = 408/496). Of the 17.7% (n = 88) involved margins, 15.9% (n = 79) of patients underwent re-excision. Of these, 7.5% (n = 37), which is 47% of all re-excisions, underwent re-excision during the planned second stage of a two-stage surgery. The remaining 8.5% (n = 42) underwent re-excision after flap insertion. Four patients who had re-excisions received neoadjuvant systemic treatment. The completion mastectomy rate was 1.8% (n = 9) due to multiple involved margins.

### 3.4 Secondary outcomes

#### 3.4.1 Revisional and symmetrisation surgery

Six patients (1.2%) required a contralateral symmetrising procedure, while 2.6% (n = 13) required corrective procedures, including lipomodelling and/or scar revision.

#### 3.4.2 Surveillance

Fourteen patients (2.9%) underwent recall biopsy due to symptoms or findings during mammographic surveillance.

#### 3.4.3 Recurrence rates and Mortality

At the median follow-up of 23 months (IQR; 11–39), the recurrence rates were as follows: local, 1% (5/504); regional/nodal, 0.6% (3/503); and distant, 3.2% (16/495). There were 11 (2.8%) mortalities out of 389 patients with recorded mortality, of which breast cancer-specific mortality was 2.1% (8/389).

## 4 Discussion

This is the analysis of the largest aggregated cohort of patients (n = 507, 2011–2021, 15 UK centres) undergoing partial breast reconstruction with a pedicled chest wall perforator flap to avoid deformity following breast conservation. Data demonstrates its applicability in T1-T2 tumours (median, 26 mm, IQR; 18–35) with re-excision rates of 15.9% and 1.8% completion mastectomy. Postoperative complication rates were in 12% of patients, including overall flap loss (0.6%, n = 3; 1 full) leading to readmissions (2.6%, n = 13) and re-operations (2.6%, n = 13). The local recurrence rate was 1% at a median follow-up of 23 months (IQR; 11–39).

83% of operations were performed in the last five years, confirming increasing CWPF practice. Non-normal distribution of cases centre-wise reflects varying uptake and experience inherent to the uptake of new techniques that may differ in a future study when individual and collective experience is more mature.

The median patient age was 54 years, and 8.1% (n = 41) were over 70 suggesting CWPF's applicability irrespective of patient age. The median BMI of 25.4 kg/m^2^ and breast size (50.4% A-B cup) suggest case selection for lower BMI with a higher tumour: breast volume ratio. Although therapeutic mammaplasty is not a comparable operation (displacement technique), in a UK multi-centre mammaplasty study [23], the median BMI was 28.3 with only 22.7% ≤ 25 (classed as normal BMI).

73.4% of patients had a breast cup size of A-C, and with T2 median tumour size of 26 mm, BCS was feasible, with most achieving clear margins. A single-centre series [24] compared tumour and specimen 3-dimensional measurements in mammaplasty (n = 31) versus flaps (n = 29). Although the anteroposterior tumour dimension in the flap was significantly lower than mammaplasty (13.6 vs 19.3 mm; p = 0.036), radial tumour dimensions were non-significantly different between the two. This supports that flaps can help achieve similar radial margin clearances (both better than simple wide local excisions).

84.7% (n = 414/489) of CWPFs were in T1-2 tumours (stages included in all breast-conserving versus mastectomy trials). To put in context, within the limitation of a non-randomised dataset with limited follow-up (median, 6.28; 0.01–11.7), a recent large (n = 48,986) Swedish cohort of T1-2 N0-2 showed worse overall and breast-cancer specific survival in mastectomy with radiotherapy (HR, 1.24; 95% CI, 1.13–1.37 and HR, 1.26; 95% CI, 1.08–1.46, respectively) or without radiotherapy (HR, 1.79; 95% CI, 1.66–1.92 and HR, 1.66; 95% CI, 1.45–1.90, respectively) [25]. The smaller proportion of patients where CWPF were performed for T3 tumours (4.3%, 21/489) may reflect the trend towards adopting extreme oncoplastic techniques for BCS [26]. These likely were due to patient preference or due to discrepancies between imaging and pathology tumour sizes, including due to DCIS (38%, 8 cases of the T3 tumours). MRI is increasingly used for preoperative assessment of DCIS. A systematic review revealed that although MRI is a more accurate predictor of actual tumour size than conventional imaging, it does not appear to translate to improved surgical outcomes (re-excisions or mastectomy) [22].

Complications were similar to those in a recent systematic review of 432 CWPFs [14]: 12.3% (haematoma 1.9%, infection 2.1%, and flap necrosis 2.1%); and to those in a systematic review of 1324 oncoplastic reduction mammoplasties [27]: 13.2% complications (wound dehiscence, 4.6%; wound infection, 2.8%; partial/total nipple necrosis, 0.9%). A collated comparative data from UK prospective studies showed 21%, 37.2% and 35.6% complication rates in therapeutic mammaplasty (n = 376), mastectomy (n = 1532) and mastectomy with immediate reconstruction (n = 1008), respectively [28]. Despite the limitations of retrospective nature and challenges of dissection around smaller perforators, the study does not reveal disproportionately higher adverse outcomes when compared with BCS and mammaplasty.

Seroma is not a complication but a sequel of the procedure [29] and yet was recorded in 3.7% (n = 19), all in the flap donor site likely due to recipient site (excision defect) space being plugged with flap. The only significant factor associated with a lack of complications was the absence of axillary surgery. This is not an unexpected finding, given the co-morbidities related to the axillary surgery [30]. However, it was impossible to disengage retrospectively, CWPF with or without axillary surgery for analysis. In AIC statistical analysis, we did not see an association between co-morbidities and smoking with complications, which prospective studies need to explore.

Margins were involved in 17.7% (n = 88), and of these, 15.9% (n = 79) had re-excisions, including 8.5% (n = 42) unplanned re-excisions and the remaining (1.8%) completion mastectomy. This is consistent with the UK 2016 margins audit (n = 2,858) that revealed a 17.2% re-operation rate, including a 2.9% mastectomy [31]. The audit extrapolated that if all the units applied SSO-ASTRO guidelines (no tumour at ink for invasive and 2 mm for DCIS), the re-excision rate would drop to 15.4%. Whether OBS and CWPF procedures in themselves and the margin distance affect the re-excision rates was explored in a small retrospective single centre/surgeon study [12]. It explored hypothetical re-excision rates between simple BCS, mammaplasty and CWPF with an assumed margin distance of 0, 1 or 2 mm to ascertain if the distance affected success. Oncoplastic BCS allowed for a 10–15% less re-excision regardless of margin policy though only significant with the 1 mm policy adopted by the UK [18]). There was no significant difference between mammaplasty and CWPF, respectively, at 2 mm (mammaplasty 15.8% vs CWPF 18.8%), 1 mm (5.3% vs 6.3%) and 0 mm (5.3% vs 6.3%). Our study period straddled the UK policy change timeline [18].

A meta-analysis [32] comparing oncoplastic breast surgery and standard BCS revealed a significantly lower re-excision rate in the oncoplastic group (RR, 0.66; 95% CI, 0.48–0.90; p = 0.009) though pooled data from nine studies showed that the total relapse rate was similar in the two groups (RR, 1.07; 95% CI, 0.88–1.30; p = 0.525). A recent Swedish population-based register study of 57,152 women with registered 1,854 major postoperative complications revealed an association with inferior survival [33]. Complication rates seen in our cohort suggest CWPF as a viable oncoplastic BCS option.

Only 1.2% of patients (n = 6) required symmetrisation surgery and 2.6% (n = 13) required lipomodelling and/or scar revision, which is in keeping with the literature [13,14]. This alone may be the most advantageous characteristic as opposed to any option that does not replace defects, i.e., the displacement option of therapeutic mammaplasty. In the absence of ptosis data, anecdotally, CWPF best applies to small-sized, non-ptotic or mildly ptotic breasts. However, some breasts will be suitable for both displacement and replacement options, with the final decision dependent on shared decision-making largely reliant on patient choice.

Radiotherapy is an integral component of BCS, and tumour bed boost radiotherapy is often used to minimise the risk of local recurrence [34]. However, data on boost radiotherapy target volumes and doses were largely unavailable, preventing an analysis of its accuracy and the impact on surgical outcomes. Since planning integrated boost after CWPF is complex and potentially prone to inaccuracy, discussion with radiation oncologists is recommended when introducing this technique in a new unit [35].

Our study is limited by its retrospective nature and medium-term follow-up. However, shorter follow-ups are not uncommon in oncoplastic breast surgery due to the recency of these procedures, as noted in the systematic reviews of flaps [14] and mammaplasty [27] with similar reported recurrence rates. Limited data on certain variables should be considered when interpreting subset analysis, such as BMI, Bra cup size or the more accurate tumour: breast ratio calculation. Bra cup size is only a subjective measure of breast size though this is the most practical tool to compare breast sizes.

Radiotherapy can affect the short- and long-term aesthetic outcomes of oncoplastic surgery by affecting the breast in multiple ways: the breast, the breast skin and the parenchyma [36]. The lack of objective and patient-reported outcome data limited our dataset, highlighting the importance of establishing a practical and prospective evaluation process using available patient-reported outcome measurement tools (PROMs). Due to a lack of specific PROMS, one centre [37] initiated the use of a combination of Breast-Q (combined BCS and LD modules) after due permission from copyright holders [38]. Later, two centres’ data [39] on 36 patients revealed 80% patient satisfaction; however, this was severely limited due to missing out on patient demographics and treatment data as these questionnaires were given and recorded anonymously with the well-intentioned avoidance of bias. Therefore, there is a need for such PROMs data.

## 5 Conclusions

Our study reflects real-life clinical practice with outcomes from different centres with variable caseloads. Complication rates within 30 days, revisional surgery and locoregional recurrence rates (at short-term follow-up) suggest CWPF as a viable oncoplastic BCS option. These flaps are an established option that should be offered to patients with a higher tumour-to-breast volume ratio and small to medium-sized non-ptotic or mildly-ptotic breasts, as shown in this world's largest cohort study on partial breast reconstruction using CWPF. In the future, our collaborative intends to track the long-term outcomes of this cohort alongside an ongoing prospective multi-centre cohort study (The PartBreCon-Pro study: PARTial BREast ReCONstruction with CWPF: PROspective study, including PROMS) that will evaluate surgical and oncological outcomes (specifically complex radiotherapy details) and collate PROMs data in centres that use Breast-Q. It should provide further useful data on partial breast reconstruction using CWPF.

## Funding

This study was borne out of voluntary multicentre collaboration with no funding.

## Ethical approval

Each hospital gained local authority approval for the data.

## Previous presentation

At the Association of Breast Surgery annual conference, May 2021: Partial breast reconstruction with chest wall perforator flaps – initial data from ‘PartBreCon’ collaborative data. Eur J Surg Oncol, 47 (2021), e286-295: e293. Abstract No. 24.

## Trial registration

Not applicable.

## Approval for study

Each hospital gained local authority approval for the data.

## Declaration of competing interest

None of the authors or collaborators declared any conflict of interest related to this project.
